# On Random Subspace Optimization-Based Hybrid Computing Models Predicting the California Bearing Ratio of Soils

**DOI:** 10.3390/ma14216516

**Published:** 2021-10-29

**Authors:** Duong Kien Trong, Binh Thai Pham, Fazal E. Jalal, Mudassir Iqbal, Panayiotis C. Roussis, Anna Mamou, Maria Ferentinou, Dung Quang Vu, Nguyen Duc Dam, Quoc Anh Tran, Panagiotis G. Asteris

**Affiliations:** 1 Faculty of Civil Engineering, University of Transport Technology, 54 Trieu Khuc, Thanh Xuan, Hanoi 100000, Vietnam; duongtk@utt.edu.vn (D.K.T.); dungvq@utt.edu.vn (D.Q.V.); damnd@utt.edu.vn (N.D.D.); 2 State Key Laboratory of Ocean Engineering, Department of Civil Engineering, Shanghai Jiao Tong University, Shanghai 200240, China; jalal2412@sjtu.edu.cn (F.E.J.); mudassiriqbal29@sjtu.edu.cn (M.I.); 3 Department of Civil Engineering, University of Engineering and Technology, Peshawar 25000, Pakistan; 4 Department of Civil and Environmental Engineering, University of Cyprus, Nicosia 1678, Cyprus; roussis@ucy.ac.cy; 5 Computational Mechanics Laboratory, School of Pedagogical and Technological Education, Heraklion, 14121 Athens, Greece; a.p.mamou@gmail.com; 6 School of Civil Engineering and Built Environment, Liverpool John Moores University, Liverpool L3 3AF, UK; M.Ferentinou@ljmu.ac.uk; 7 Department of Civil and Environmental Engineering, Norwegian University of Science and Technology, 7491 Trondheim, Norway; quoc.a.tran@ntnu.no

**Keywords:** California Bearing Ratio, modulus of subgrade reaction, elastic modulus, metaheuristic algorithms

## Abstract

The California Bearing Ratio (CBR) is an important index for evaluating the bearing capacity of pavement subgrade materials. In this research, random subspace optimization-based hybrid computing models were trained and developed for the prediction of the CBR of soil. Three models were developed, namely reduced error pruning trees (REPTs), random subsurface-based REPT (RSS-REPT), and RSS-based extra tree (RSS-ET). An experimental database was compiled from a total of 214 soil samples, which were classified according to AASHTO M 145, and included 26 samples of A-2-6 (clayey gravel and sand soil), 3 samples of A-4 (silty soil), 89 samples of A-6 (clayey soil), and 96 samples of A-7-6 (clayey soil). All CBR tests were performed in soaked conditions. The input parameters of the models included the particle size distribution, gravel content (G), coarse sand content (CS), fine sand content (FS), silt clay content (SC), organic content (O), liquid limit (LL), plastic limit (PL), plasticity index (PI), optimum moisture content (OMC), and maximum dry density (MDD). The accuracy of the developed models was assessed using numerous performance indexes, such as the coefficient of determination, relative error, MAE, and RMSE. The results show that the highest prediction accuracy was obtained using the RSS-based extra tree optimization technique.

## 1. Introduction

Accurate prediction of the mechanical index of geomaterials is critical for robust pavement design [1,2]. The strength of the subgrade soil is routinely assessed in terms of its California Bearing Ratio (CBR). The California Bearing Ratio (CBR) of soil is a static strength and bearing capacity index, which may be obtained from either laboratory or in situ measurements [3,4]. The CBR is an important input parameter predicting the stiffness modulus of the soil subgrade, which is a key pavement design index considering the effect of cyclic loading on the soil’s stiffness [5,6,7]. The CBR value is used to indirectly estimate the thickness of the subgrade materials in major infrastructure projects. Therefore, fast and reliable estimation of this parameter is significant to the design process and relevant construction time.

The CBR test was originally introduced by the California Highway Department, during World War-II, and was subsequently adopted widely as a standard method for soil strength and bearing capacity evaluation [8], obtained using either ASTM Standard D-1883-05 or BS 1377 [9]. Laboratory tests are performed on compacted soil samples with OMC in un-soaked and soaked conditions, and they can also be carried out on natural soils. According to current AASHTO 2003 standards, the laboratory CBR test involves soil mass penetration using a circular 50mm diameter plunger applied at a 1.25 mm/min rate [10] into a compacted soil specimen at the optimum moisture content. The in situ CBR tests are conducted at the natural ground surface level, prepared subgrade level, or on a level surface of the test pit at the construction site. The applied plunger pressure is then divided by the pressure that is required to incur the same penetration of standard crushed rock [11]. The CBR value of soil is influenced by a number of parameters, namely the particle size, soil fabric, plasticity index, moisture content, suction, and dry density [11,12].

Direct laboratory or in situ measurement of the CBR index of soil is a lengthy process, often yielding inaccurate results due to the disturbance of samples, negligence during testing, and poor testing facilities. In this context, developing artificial intelligence (AI) models for the prediction of CBR may be a viable alternative [1], if CBR could be obtained through a cost-effective method resulting in less construction time. Artificial intelligence models can simulate highly non-linear associations between numerous input and output parameters and can therefore provide more accurate predictions than those obtained using simple and multiple regression analysis [13,14,15]. During the last decade numerous artificial intelligence models techniques, including artificial neural network (ANN) [16], adaptive neuro-fuzzy inference system (ANFIS) [17], gene and multi expression programming [18,19,20], ensemble framework techniques (for instance, bagging, rotation forest, and random subspace (RSS)) [21], decision tree (DT) [22], and support vector machine (SVM) [23], have been used in engineering and numerous other disciplines [13,24]. Hybrid ensemble strategies including bagging, RSS, and boosting with ensemble pruning are particularly suitable for extracting deep features from multivariate data [25].

The aim of this research was to develop subspace optimization-based hybrid computing models for the prediction of CBR using 10 input variables: gravel percentage (G), coarse sand % (CS), fine sand % (FS), fine material (silt and clay %—passing sieve No. 200) (SC), organic matter content (O), liquid limit (LL), plastic limit (PL), plasticity index (PI), optimum moisture content (OMC), and maximum dry density (MDD). To this end, three hybrid ensemble models were developed, i.e., REPT (reduced error pruning trees), RSS-REPT, and RSS-ET (RSS-extra trees). The accuracy of the models was assessed using numerous performance indexes, such as the root mean square error (RMSE), mean absolute error (MAE), and correlation coefficient (R^2^).

## 2. Short Literature Review on Soft Computing Techniques for Estimation of the California Bearing Ratio

The story of predictive models based on available test data to forecast the CBR value can be traced back to Black (1962) [26], who developed a correlation scheme for CBR estimation of cohesive soils based on index properties, such as the plasticity index (PI) and liquidity index (LI). Johnson and Bhatia in (1969) [27] suggested a correlation for CBR based on the suitability index, which relies on plasticity and particle size distribution data from lateritic gravel soils in Ghana. Agarwal and Ghanekar in (1970) [28], based on experiments on fine-graded soils, proposed a correlation between CBR and Atterberg limits. Yet, the authors suggested that poor correlations were revealed among the input parameters, while only OMC and LL were found to be significant; therefore, the applicability of the method was limited to preliminary soil identifications.

Later, Al-Refeai and Al-Suhaibani (1997) [29], relying on data obtained from different soils ranging from clay to gravely sand, from dynamic cone penetrometer tests, suggested a relationship between the moisture content, dry density, and penetration depth with CBR, and performed tests for a range of moisture content and density conditions.

Stephens in (1992) [30], based on series of data from the Natal Roads Department in Pietermaritzburg, South Africa, reviewed the performances of earlier published models and commented that most of them were unsatisfactory for universal use, particularly for problematic soils like expansive and shrinking soils, widely exposed in South Africa. One of the key issues identified was the impact of the clay fraction determination on CBR value estimation. The author proposed the use of shrinkage properties and gradation to allow for CBR estimation for shrinking and non-shrinking soils.

In the USA, under the National Cooperative Highway Research Program (NCHRP) (2004) [31], two empirical predictive models were suggested, using the index properties of soil for clean (i.e., fine content ≥ 12%) and coarse-grained soils. Thereafter, Kin in (2006) [32] reviewed the correlation equations given by NCHRP and found limitations in CBR estimation for coarse-grained soils, whereas for fine-grained soils, the NCHRP models were found to be of moderate accuracy.

During the last decade, many authors used statistics, proposing simple and multiple regression predictive models for CBR value estimation, based on gradation analysis, Atterberg limits, optimum moisture content, and maximum dry density among others [1,3,33,34,35,36,37,38].

A close review of the literature presented from Black (1962) [26] of the latest studies suggests that the prediction of CBR values from presented and reviewed statistical models is generally problematic, has poor applicability and potential for universal models, and is likely to be limited to the local datasets. This is partly attributed to the small number of observations available used in the predictive models, the complexity and non-linearity of the problem itself, the uncertainty involved in the determination of soil properties, the scatter of data, and the particulate nature of soil, which is seldom elastic, isotropic, or homogeneous.

Machine learning (ML) is an area of computational mechanics that sufficiently handles complex problems, exposing non-linear characteristics, including a high level of uncertainty as evidenced by recently published studies in the broader spectrum of geotechnical engineering. Advanced ML techniques, which are more competent in non-linear modelling, provide a feasible tool for simulating several multifaceted processes [39].

Several AI-based models have been used in the last decade to predict the CBR value of soils. Various biologically inspired algorithms, such as artificial neural network (ANN), support vector machines (SVMs), gene expression programming (GEP), generalized regression neural networks (GRNNs), multi-layer perceptron neural networks (MLPNs), and group method of data handling (GMDH), have been exploited and produced promising results, as shown in Table 1.

Taskiran (2010) [40] proposed an ANN and a GEP model to forecast the CBR value of fine-grained soils. The compiled data set included 151 CBR test data for soils classified as A-4 to A-7. The performance of the models was found to range (R^2^ > 0.90) for both the models.

Yildirim and Gunaydin (2011) [41], Kumar et al. (2013) [42], Varghese et al. (2013) [43], Bhatt and Jain (2014) [34], Sabat (2015) [44], Ghorbani and Hasanzadehshooiili (2018) [45], Suthar and Aggarwal (2018) [33], Alam et al. (2020) [46], and Islam and Roy (2020) [47] developed further models relying on limited data sets (in the range of 20 to 158 observations). Their findings suggested accuracies of 0.81 R^2^ < 1.00. Later SVM models developed by Sabat (2015) [44], using 49 CBR test data of stabilized soils, were found to show a performance of R^2^ = 0.96. Only recently, Taha et el. (2019) [48] used larger data sets of 218 laboratory tests and developed an ANN-based model with a prediction accuracy of R^2^ = 0.88. In addition, Tenpe and Patel (2020) [49] used 389 soil test data and produced two models using SVM and GEP algorithms, with a performance accuracy ranging between 0.83 < R^2^ < 0.90. Al-Busultan et al. (2020) [50] used a dataset of 358 tests and developed an ANN model with an R^2^ = 0.78.

The prediction capability of the presented models, as was quantified through R^2^ metrics, suggests the paradox of higher predictive accuracies based on predictive models developed using smaller datasets, compared to predictive models that used larger data sets and yielded comparatively lower R^2^ values of moderate accuracy [49,50]. This is most probably a result of overfitting and network memorizing of the particular local dataset, which results in the models being weak in generalization. It is very common for back-propagation-based models to become trapped in local minima, leading to erroneous results [51]. It is important to highlight that studies based on larger training data sets include a more representative description of the specific geotechnical problem, and therefore are expected to be more reliable for future predictions. The reliability of a model depends on the comprehensiveness of the input data set. The incorporation of a wide variety of soils, as per the Unified Soil Classification System, which covers a range of engineering properties that affect the stiffness of a soil, such as soil index properties and particle size distribution, satisfies the criteria for a promising prediction model.

To address these deficiencies, scholars have proposed hybrid models by integrating optimization algorithms (OAs) and common soft computing models to search for the exact global minimum instead of finding the local minima [47,52]. Hybridization of OAs and CSC techniques balances the exploration and exploitation processes and generates optimized values of learning parameters (weights and biases), which in turn are used to enhance the performance of CSC techniques.

Bradhan et al., 2021a [53] and Bradhan et al., 2021b [54] proposed a novel integration of extreme learning machine (ELM) and adaptive neuro swarm intelligence (ANSI) techniques for the determination of the California Bearing Ratio (CBR) of soils and the results were prominent.

Onyelowe et al., 2021 [16] applied evolutionary hybrid algorithms of ANN, Levenberg–Marquardt back-propagation (LMBP), Bayesian programming (BP), and conjugate gradient (CG) algorithms to predict the CBR value of ash-treated expansive soil, and the correlation was found to be R^2^ = 0.9.

Raza et al., 2021 [55], identified a gap in the literature in the prediction of geosynthetic-reinforced subgrade soil, and used data-driven-based machine learning models to estimate the CBR value. Several intelligent models, such as artificial neural network (ANN), least median of squares regression, Gaussian processes regression, elastic net regularization regression, lazy K-star, M-5 model trees, alternating model trees, and random forest, were proposed and the prediction accuracy was found to be 0.80 < R^2^ < 0.92.

**Table 1:** Prediction accuracy of the soft computing models predicting the CBR of soil reported in the reviewed literature.

Reference	Model(s) Employed	Prediction Accuracy	Total Nr of Data/Types of Soils
Bardhan et al., 2021a [53]	MARS-L	R^2^ = 0.96 RMSE = 0.0359	362
Bardhan et al., 2021b [54]	extreme learning machine (ELM)-based models	0.81< R^2^ <0.91	312
Onyelowe et al., 2021 [16]	Levenberg–Marquardt backpropagation (LMBP), Bayesian programming (BP), and conjugate gradient (CG)	R^2^ = 0.90	129
Raza et al. 2021 [55]	least median of squares regression, Gaussian processes regression, elastic net regularization regression, lazy K-star, M-5 model trees, alternating model trees, and random forest	0.80 < R^2^ <0.92	97 tests

In the current study, the proposed hybrid model aimed to develop subspace optimization-based hybrid computing models for the prediction of CBR using 10 input variables: gravel % (G), coarse sand % (CS), fine sand % (FS), fine material (silt and clay %—passing sieve No. 200) (SC), organic matter content (O), liquid limit (LL), plastic limit (PL), plasticity index (PI), optimum moisture content (OMC), and maximum dry density (MDD), using a comprehensive data set of 214 CBR tests of various soil types.

## 3. Materials and Methods

### 3.1. Database for the Training of Soft Computing Models

The reliability of the database used to train soft computing models is undoubtedly a critical parameter affecting the reliability of the actual model prediction. Interestingly though, the reliability of the actual database used to train soft computing models has generally received less attention than, for example, the model architecture and the various transfer functions used. A reliable database should not only comprise a statistically significant amount of representative data, but the data distribution should also comply with fundamental statistical analysis principles and the experimental/field data should be reported in compliance with international standards.

**Figure 1:**
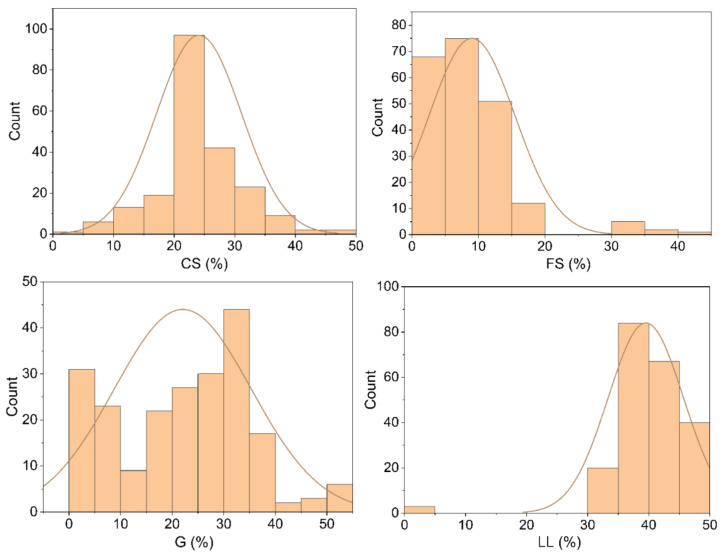

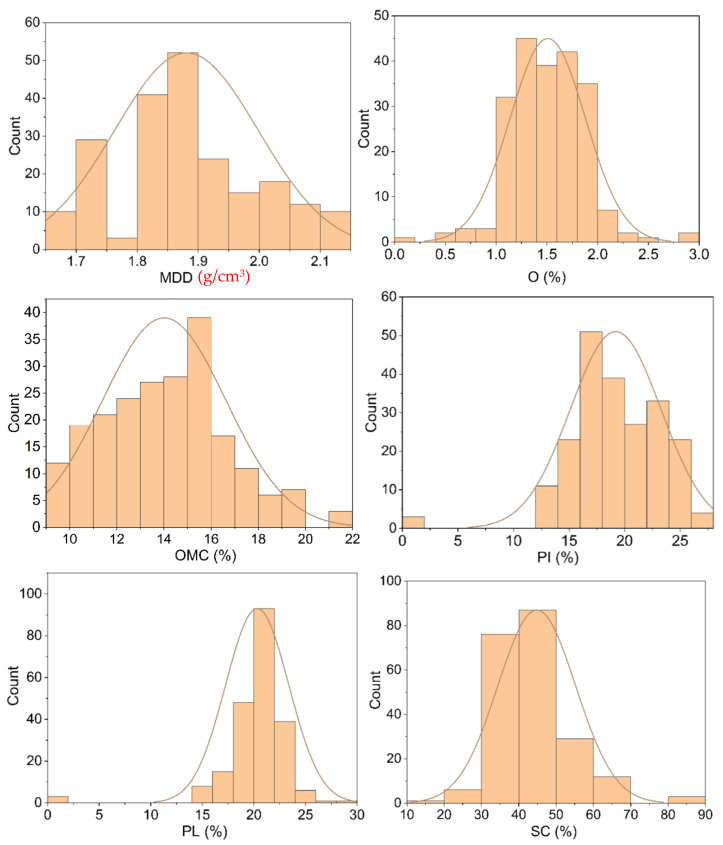
Statistical analysis distribution of the input parameters in this study.

**Figure 2:**
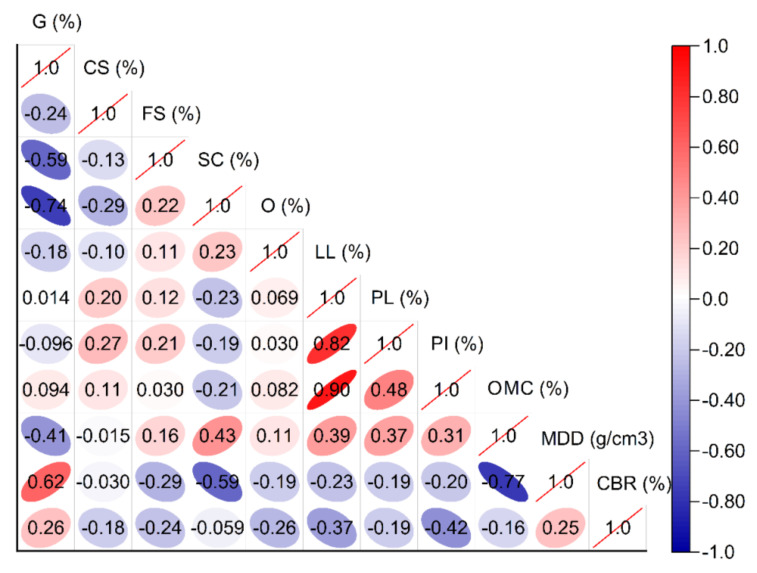
Correlation matrix analysis input variables in this study.

In light of the above, an experimental database was compiled from samples collected at the Van Don - Mong Cai expressway project, in the Quang Ninh province of Vietnam. The starting point of the route is located at 70 + 108 km and intersects the endpoint of the main road connecting the Van Don zone in the Doan Ket commune, the Van Don District, and the Quang Ninh province. The endpoint of the route is located at 150 + 338 km and intersects the 335 provincial road, at the starting point of the Bac Luan 2 Bridge Path project in the Hai Hoa ward, Mong Cai city of the Quang Ninh province [56]. A total of 214 samples were collected during the period spanning from November 8, 2019 to July 1, 2021. The soil samples were then transferred to the laboratory, whereupon the particle size distribution (AASHTO T 88 [57] and ASTM D 422 [58]), liquid limit (AASHTO T 89 [59] and ASTM D 4318 [60]), organic content (AASHTO T 267 [61]), compaction curves (ASTM D 4253 [62] and ASTM D 4254 [63] [64]), and the CBR of the soil were determined (Figure 1 and Figure 2). The statistical parameters, such as the minimum, average, maximum, and standard deviations, are presented in detail in Table 2.

The California Bearing Ratio (CBR) relates the penetration resistance of laboratory-compacted soil material to that of well-graded (poorly sorted), durable, and crushed rock material [65]. The CBR was developed by the American Society for Testing and Materials [66] in North America [65] and the American Association of State Highway and Transportation Officials [67] for assessing the penetration resistance of subbase and subgrade pavement materials. The test involves compaction of the soil in a standard mold size (177.8 mm height and 152.3 mm diameter). The moisture content and compaction energy may vary with individual project’s requirements. The load is applied through a 49.6 mm diameter steel piston at a 1.3 mm penetration rate per minute. The load required to incur a 2.54 mm and 5.08 mm penetration is continuously measured and converted to stress by dividing it with the area of the steel piston. The CBR is then calculated as the ratio of the required laboratory stress over the corresponding crushed aggregate standard penetration load [65]. A minimum CBR of 10 is generally required for subgrade design [64].

The CBR of soil is influenced by a number of parameters including, for example, the particle size, soil fabric, plasticity index, moisture content, suction, and dry density [11,12]. In the laboratory, the CBR is determined as the in situ moisture content and corresponding dry density. Whilst the in situ dry density of the soil can be determined with reasonable accuracy, determining the in situ moisture content may be challenging. In general, as the moisture content is reduced and the suction increases, the soil shifts from a bulk-water-regulated to a menisci-water-regulated response and the CBR is reduced significantly at the wet side of the optimum [68,69]. In this research, the CBR at the equilibrium moisture content (4 days soaked CBR) was measured [70].

### 3.2. Sensitivity Analysis of the Input Parameters Predicting the CBR of Soil

A sensitivity analysis was performed to identify which of the 10 input parameters (gravel percentage (G), coarse sand percentage (CS), fine sand percentage (FS), fine soil material (silt clay percentage) (SC), organic matter content (O), liquid limit (LL), plastic limit (PL), plasticity index (PI), optimum moisture content (OMC), and maximum dry density (MDD)) significantly affected the predicted CBR of soil. The aim of the sensitivity analysis was to remove the input parameters that have the smallest influence on the predicted output parameter, thereby significantly reducing the required complexity and training time of the model. In this research, the cosine amplitude method (CAM) was used to perform the sensitivity analysis [71,72]. In CAM, data pairs are used to construct a data array, X, as follows:

(1) $$X=\left\{x_{1},x_{2},x_{3},\ldots ,x_{i},\ldots ,x_{n}\right\}$$

where the $x_{i}$ variable, in the X array, is a m length vector, which may be expressed as:

(2) $$x_{i}=\left\{x_{i1},x_{i2},x_{i3},\ldots ,x_{\mathrm{im}}\right\}$$

The relationship between $R_{\mathrm{ij}}$ (strength of the relation) and the $x_{i}$ and $x_{j}$ datasets may be expressed as:

(3) $$R_{\mathrm{ij}}=\frac{\sum_{k=1}^{m}x_{\mathrm{ik}}x_{\mathrm{jk}}}{\sqrt{\sum_{k=1}^{m}x^{2}{}_{\mathrm{ik}}\sum_{k=1}^{m}x^{2}{}_{\mathrm{ik}}}}$$

**Figure 3:**
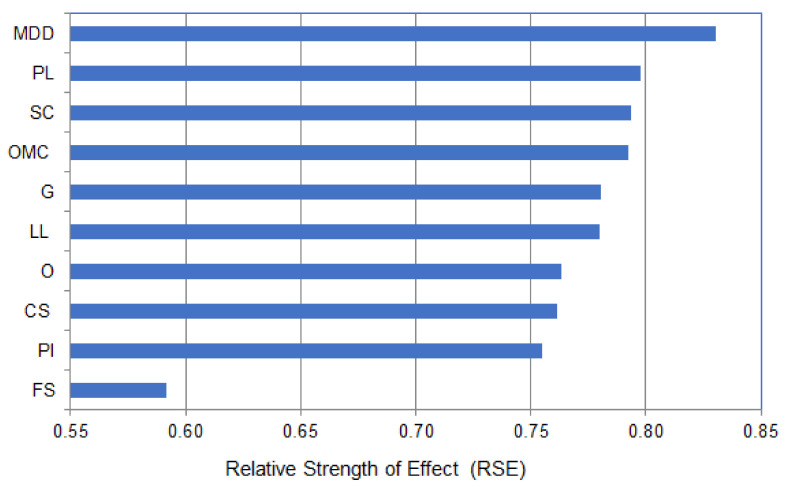
Sensitivity analysis of the input parameters predicting the CBR of soil.

The results of the sensitivity analysis presented in Figure 3 show that the highest and smallest relative strength effect (RSE) on the CBR of soil was obtained for the maximum dry density (MDD)) (RSE = 0.8301) and the fine sand percentage (FS) (RSE = 0.5915), respectively. The other eight input parameters registered moderate RSE values ranging between 0.7548 and 0.7974.

### 3.3. Methods Used

**Figure 4:**
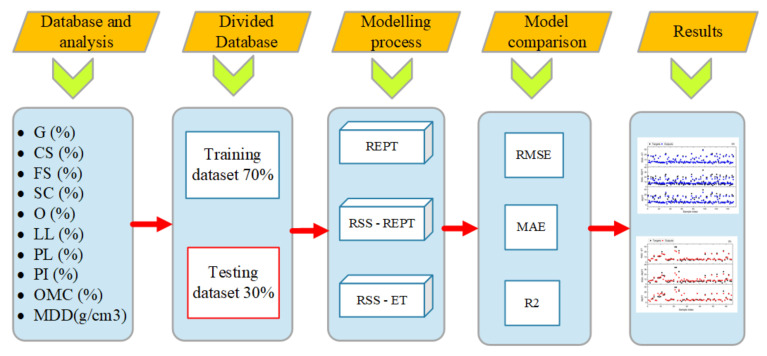
Methodological framework for the predicted values in this study.

This section presents the methodology used to train and develop the soft computing models. A database comprising 10 input parameters: gravel percentage (G), coarse sand percentage (CS), fine sand percentage (FS), fine soil material (silt clay percentage) (SC), organic matter content (O), liquid limit (LL), plastic limit (PL), plasticity index (PI), optimum moisture content (OMC), and maximum dry density (MDD), for the prediction of the soil’s CBR was compiled (Figure 4). The database was split into training and testing datasets at a ratio of 70% to 30%. For the modelling of the California Bearing Ratio (CBR), three soft computing models were trained and developed, such as REPT, RSS-REPT, and RSS-ET, and their accuracy was evaluated using a variety of performance indexes, such as RMSE, MAE, and R^2^. The RSS-REPT and RSS-ET are hybrid models, which were developed using a combination of RSS ensemble techniques and two predictors, namely REPT and ET. In the hybrid models, RSS ensemble was firstly used to optimize the training dataset, and then the optimal training dataset was used to train the predictors (REPT and ET). The Weka software was used as a platform for training and validating the models. Detailed and in-depth background theory of the methods used herein is presented in the following sections.

#### 3.3.1. Random Subspace (RSS)

The random subspace method (RSS) is a random sampling ensemble method used to produce different representations that could be employed in generating a variety of decision agents [73,74]. A typical RS model comprises an integrated algorithm that establishes a DT based on a classifier supporting the maximum accuracy in the case of training data. This method is used to improve the performance of weak classifiers [75]. Thereafter, the RSS incorporates randomness inside the problem representation by randomly choosing specific variables that are to be replaced [74]. According to Plumpton et al., [76], the RS approach is an efficacious ensemble and it exhibits many diverse classifiers since it combines the accuracy of the weak classifiers [77]. Moreover, it resembles the bagging algorithm in terms of stochastic discrimination theory as a random selection is made by the original training set [78]; however, the RSS is chosen using the original training set of characteristics [79]. 

This technique has been applied to a significant number of nonlinear problems [78], in various disciplines (medical science, computer science, and banking). The application of the RSS technique in transportation engineering is still limited [73]. A detailed presentation of the RSS technique is as follows:

It is assumed that X = [x_1_, x_2_, …, x_n_] refers to a vector of n number of affecting parameters. Constructing an RSS ensemble to consolidate various classifiers for cataloging purposes, N samples having a size of Z are arbitrarily selected using a uniform distribution over X so that no replacement is required. Every specimen depicts the associated individual subset that expresses a subspace of X. After that, the training of a classifier takes place considering either a single subset or a whole training set [73]. However, the aforementioned amendment is done in the feature space (instead of the instance space). The pseudo-code in the case of the RSS algorithm (Algorithm 1) is reported by [80].
**Algorithm 1.** RSS algorithm  ***Input***
*: Data set D = [(x_1_,y_1_), (x_2_,y_2_),…, (x_n_,y_n_),)]*
 *  Base classifier algorithm L;*
 *  Number of subspace rate k;*
 *  Number of learning rounds T.*
 ***Process:***

 *For, t = 1,2, …, T*
 *D_t_ = RS (D,k);   % Random generate a subspace sample from D*
 *H_t_ = L(D_t_);    % Train a base  classifier h_t_ from the subspace sample*
 *End.*
 ***Output:***
*H(x) = *
$\arg\;max_{y\epsilon x}\sum_{t=1}^{T}1\left(y=h_{t}\left(x\right)\right)$
*; % the value of 1(α) is 1 if α is true*
*                           % and 0 otherwise*


If the dataset exhibits a variety of redundant or irrelevant parameters, then the reliable base classifiers could be attained in random subspaces in contrast to the original feature space [80].

#### 3.3.2. Reduced Error Pruning Trees (REPT)

The reduced error pruning tree (“REPT”) is a mixture of the reduced error pruning (REP) and the decision tree (DT) algorithm technique, which comprises various splits and pruning steps. In this research, the DT was implemented to simplify the modeling process and the REP was incorporated to reduce the complexity of the tree structure. In addition, the REPT uses the validation dataset to accurately predict the generalization error [81,82]. It is important to mention that the pruning phenomenon involved in the REPT algorithm is attributed to the backward over-fitting issue. The REPT algorithm intends to search for the minimal version of the excellent sub-tree on the basis of the post-pruning technique [83]. 

The aim of the REPT is to reduce the level of modeling complexity when dealing with numerous input data. According to Pham, Jaafari, Nguyen-Thoi, Van Phong, Nguyen, Satyam, Masroor, Rehman, Sajjad and Sahana [82], the REPT technique has been used by numerous researchers to determine an optimal subtree by using the post-pruning technique. The REPT refers to a robust DT learning, such that it establishes a DT on the basis of information gain or variance reduction [84]. The performance of the REPT model is either associated with the information gain obtained from entropy or reducing the variance (as shown in Equation (4) below) and reduced error pruning methods [83]:

(4) $$\mathrm{Gain}\;\mathrm{Ratio}(x,\;Z)=\frac{Entropy(Z)-\sum_{i=1}^{n}\frac{\left|Z_{i}\right|}{\left|Z\right|}Entropy(Z_{i})}{-\sum_{i=1}^{n}\frac{\left|Z_{i}\right|}{\left|Z\right|}\log_{2}\frac{\left|Z_{i}\right|}{\left|Z\right|}}$$

where attribute x is attributed to a training dataset Z with subsets Z_i_, i = 1, 2, …, n.

Moreover, REPT may be used to reduce the size of DTs by reducing the complexity of the final classifier. The REPT also increases the degree of estimation accuracy of the classifier since it controls the over-fitting problem alongside the removal of the tree sections that tend to create noisy or erroneous data [85].

Usually, two different techniques are used for pruning the DTs by applying the information gain ratio, i.e., (i) pre-pruning and (ii) post-pruning [83]. Pre-pruning is applied when the number of instances falls below the training set percentage, signifying that this node is aggregated. Post-pruning is used when the DT has been developed to a point such that no problem is encountered in the training set [82]. While comparing the two aforementioned approaches, it is obvious that pre-pruning has the advantage of producing trees faster, while post-pruning has the capability to generate more effective trees [86]. Pre-pruning occurs when the tree expansion is stopped during the data building process. The main advantage of the REPT technique is the reduction of the complexity of the DT structure, thus avoiding the over-fitting issue in the process of learning such that the accuracy degree is not affected [87].

#### 3.3.3. Extra Tree (ET)

Introduced by Geurts, et al. [88], extra trees (ET) are also known as extremely randomized trees [89], and are essentially an extension of random forest (RF) regression and they incorporate stochasticity in the induction generation of classical DTs, thus forming a more computationally robust AI algorithm. Furthermore, ETs are considered to be the evolutionary version of the RF, yielding good results while simulating complex problems. Note that both these models comprise a series of regression tree models that are formed independently [90,91]. The ETs are different from the RF regression in terms of selecting data to train the model (ET utilizes the whole data while RF uses only a bootstrap replica) and picking the optimal feature for splitting the note (ET picks a much better feature than that of RF). Moreover, ET comprises three main governing parameters: (i) K is the number of randomly chosen variables in order to disintegrate a node, n_min_ depicts the minimum number of specimens needed for splitting an internal node, and M is the number of trees developed inside the model [91]. 

Multiple DTs are utilized, which accomplish classification as well as regression processes. The feature bagging-based split occurs in two major stages. First, the random subset of features is chosen out of the previously chosen training data subset. After that, in the second stage, the excellent subset feature alongside its corresponding value is selected for performing the decision split. Generally, the most appropriate and robust feature is chosen on the basis of Gini criteria or information gain [92].

#### 3.3.4. Prediction Accuracy Indicators

In this research, the following three performance indicators were used to assess the prediction accuracy of the developed models: the root mean square error (RMSE) [93,94,95,96], mean absolute error (MAE) [19,97,98], and correlation coefficient (R^2^) [16,97,98]:

(5) $$R=\frac{\sum_{i=1}^{m}\left(y_{cai}-\bar{y_{ca}}\right)\left(y_{mei}-\bar{y_{me}}\right)}{\sqrt{\sum_{i=1}^{m}{\left(y_{cai}-\bar{y_{ca}}\right)}^{2}{\left(y_{mei}-\bar{y_{me}}\right)}^{2}}}$$

(6) $$RMSE=\sqrt{\frac{1}{n}\sum_{i=1}^{m}{\left(y_{cai}-y_{mei}\right)}^{2}}$$

(7) $$MAE=\frac{1}{m}\sum_{i=1}^{m}\left|y_{cai}-y_{mei}\right|$$

## 4. Results and Discussion

### 4.1. Prediction Accuracy

**Figure 5:**
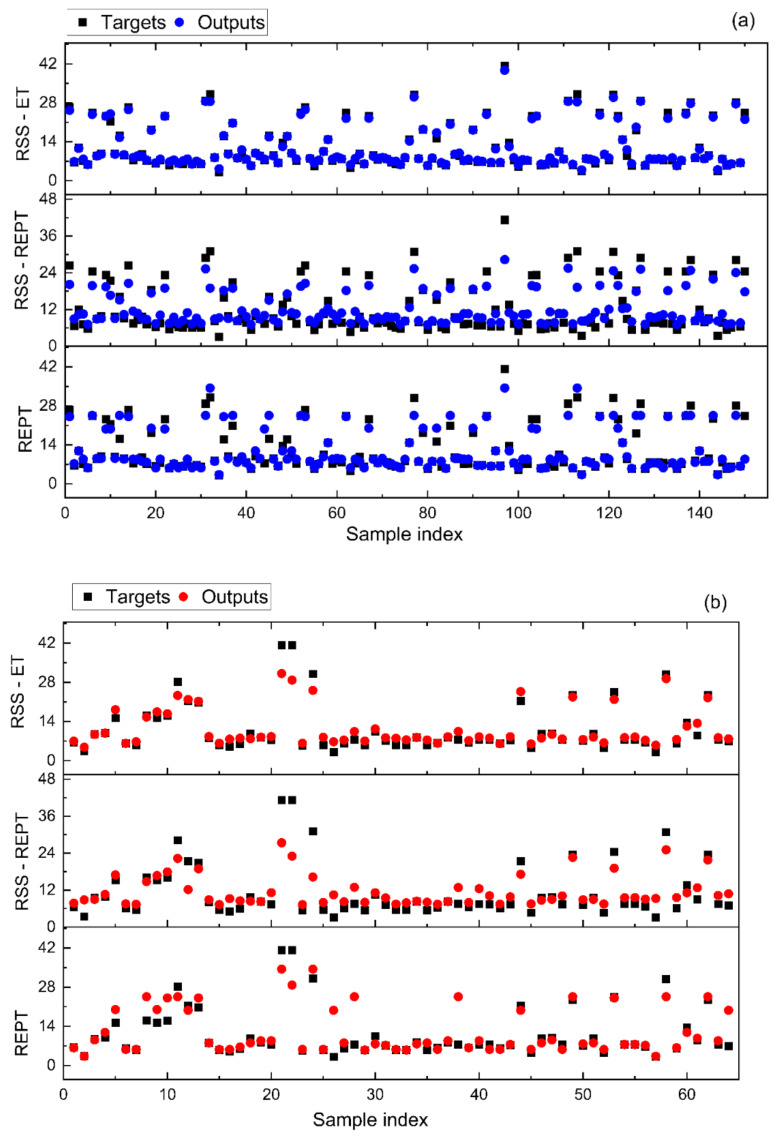
Comparison of the predicted and actual results: (**a**) Training and (**b**) test data.

Figure 5 shows the comparison of the predicted and measured California Bearing Ratio for the three different models. The correlation coefficient R^2^ of the various models during the training stage was 0.937, 0.939, and 0.995 for the REPT, RSS-REPT, and RSS-ET models, respectively (Table 3). Correlation coefficient values in excess of 0.8 are generally considered to establish a close agreement between the measured and predicted values [16,17,18,93,99,100,101,102,103]. However, the prediction accuracy of the testing dataset dropped significantly to R^2^ = 0.709 and R^2^ = 0.783 for the REPT and RSS-REPT models, respectively. Whilst these correlation coefficient values are greater than 0.8, they may indicate overfitting issues. The correlation coefficient of the REPT-ET model during the testing stage was R^2^ = 0.968, establishing the robustness of this model as compared to the REPT and RSS-REPT models. No indication of overfitting issues occurred for the RSS-ET model, which registered similar correlation coefficient values during the training and testing stage. 

**Figure 6:**
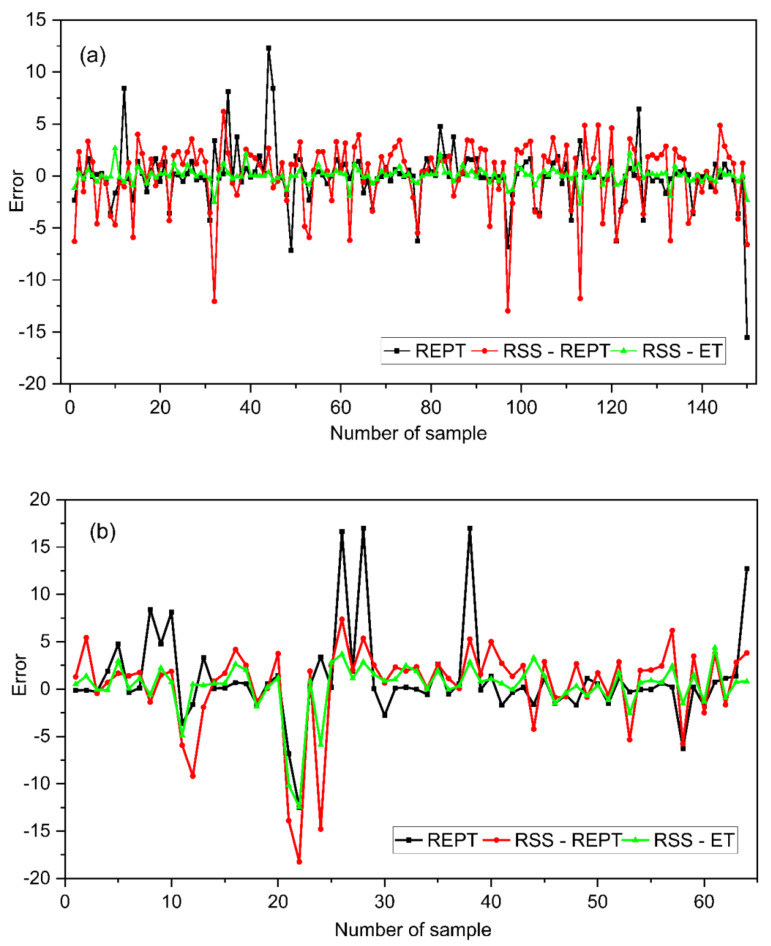
Error values for (**a**) training and (**b**) testing.

The relative error of the developed models generally ranges between ±12.5 for the training and ±17.5 for the testing dataset (Figure 6). The RSS-ET outperformed both the REPT and RSS-REPT models in terms of prediction accuracy. The error for the RSS-ET model denoted by the green line for both the training and testing datasets is the smallest. Table 3 summarizes the prediction accuracy of the models using a variety of performance indexes. The correlation and error analysis data establish the suitability of the RSS-ET model in predicting CBR values within the input data range to which the model was trained and developed. 

### 4.2. Comparison of Developed Models

**Figure 7:**
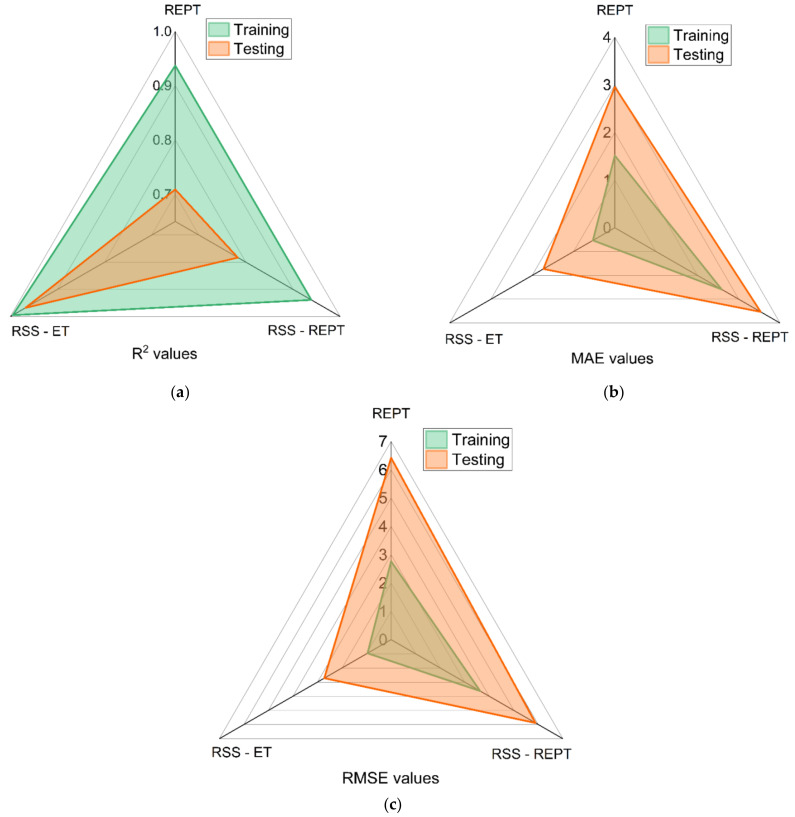
Comparative analysis of R^2^ (**a**), RMSE (**b**), and MAE (**c**) of the developed models.

Figure 7 shows the accuracy of the developed models during the training and testing stage using the root mean square error (RMSE), mean absolute error (MAE), and correlation coefficient (R^2^). The results show that the random subspace-based extra tree (RSS-ET) model outperforms the random subspace-based (RSS-REPT) and reduced error pruning tree (REPT) models regardless of the performance index is used. Moreover, the prediction accuracy of the random subspace-based extra tree (RSS-ET) model (R^2^ = 0.968) developed in this research is higher than the prediction accuracy of the soft computing models currently reported in the literature [49,104,105,106,107].

## 5. Limitations

The proposed random subspace-based extra tree (RSS-ET) neural network can predict the CBR of soil, strictly within the range of parameter values used to train and develop it, which are presented in Table 2. The predictive accuracy of the optimum RSS-ET model may also be affected by the distribution of the parameter values used for training and development. For example, the available fine sand content data are particularly limited within the 20–30% range. As part of the ongoing research, the authors aim to enrich the parameter value range, where a limited amount of data is available and to calibrate the developed random subspace-based extra tree (RSS-ET) over the enriched experimental database.

## 6. Conclusions

In this research, reduced error pruning trees (REPTs), random subsurface-based REPT (RSS-REPT), and RSS-based extra tree (RSS-ET) models were trained and developed for the prediction of the CBR of soil. The input parameters of the models include the gravel content (G), coarse sand content (CS), fine sand content (FS), silt clay content (SC), organic content (O), liquid limit (LL), plastic limit (PL), plasticity index (PI), optimum moisture content (OMC), and maximum dry density (MDD). The following main conclusions may be drawn:

1. The results show that the random subspace-based extra tree (RSS-ET) model outperformed the random subspace-based REPT (RSS-REPT) and reduced error pruning tree (REPT) models independent of which following performance indices were used: root mean square error (RMSE), mean absolute error (MAE), and correlation coefficient (R^2^).
2. The accuracy of the developed random subspace-based extra tree (RSS-ET) model to predict the CBR of soil was R^2^ –0.968 and is higher than the prediction accuracy of the soft computing models currently reported in the literature (Table 1) [16,53,54]. Whilst this is a significantly high prediction accuracy, it is strictly associated with the database used in this research. As part of ongoing research, the authors aim to enrich the parameter value range where a limited amount of data is available and to calibrate the developed random subspace-based extra tree (RSS-ET) over the enriched experimental database.
3. During the testing stage, the correlation coefficient (R^2^) values of the REPT and RSS-REPT models were significantly smaller than those obtained during the training stage, indicating overfitting issues. No indication of overfitting issues was observed for the RSS-ET model, which registered similar correlation coefficient values during the training and testing stage.

## Figures and Tables

**Table 2:** Statistical analysis of the input and output parameters.

Variable	Symbol	Units	Category	Statistics			
				Min	Average	Max	STD
Gravel content	G	%	Input	0	51.4	24.75	13.295
Coarse Sand content	CS	%	Input	3	46.3	23.7	7.017
Fine Sand content	FS	%	Input	2.5	41.5	7.25	6.468
Silt-Clay content	SC	%	Input	17.87	88.7	44.55	10.447
Organic content	OC	%	Input	0.12	2.94	1.51	0.373
Liquid Limit	LL	%	Input	2.08	48.45	39.99	6.173
Plastic Limit	PL	%	Input	1.17	28.49	20.835	3.068
Plasticity Index	PI	%	Input	0.91	27.48	18.435	4.078
Optimum Moisture Content	OMC	%	Input	9.3	21.5	14.275	2.619
Maximum Dry Density	MDD	g/cm^3^	Input	1.672	2.14	1.871	0.118
California Bearing Ratio	CBR	-	Output	3.09	41.26	7.95	8.175

**Table 3:** Statistical analysis results stemming from the current study.

No	Parameters	Training	Testing
R^2^			
1	REPT	0.937	0.709
2	Randomsubspace—REPT	0.939	0.783
3	Randomsubspace—Extra Tree	0.995	0.968
MAE			
1	REPT	1.515	2.956
2	Randomsubspace—REPT	2.586	3.534
3	Randomsubspace—Extra Tree	0.530	1.728
RMSE			
1	REPT	2.765	6.424
2	Randomsubspace—REPT	3.630	5.885
3	Randomsubspace—Extra Tree	0.960	2.725

## Data Availability

The data presented in this study are available on request from the corresponding author.

## Abbreviations

ANN	Artificial neural network
ANFIS	Adaptive Neuro-Fuzzy Inference System
CBR	California Bearing Ratio
CS	Coarse Sand
DT	Decision Tree
FS	Fine Sand
G	Gravel content
GEP	Gene Expression Programming
GRNN	Generalized Regression Neural Networks
GMDH	Group Method of Data Handling
LL	Liquid Limit
MAE	Mean absolute error
MDD	Maximum Dry Density
MLPN	Multi-Layer Perceptron Neural Networks
ML	Machine learning
O	Organic content
OMC	Optimum Moisture Content
PL	Plastic Limit
PI	Plasticity Index
REPT	Reduced Error Pruning Trees
RMSE	Root Mean Square Error
RSS	Random Subspace
RSS-REPT	Random Subspace based Reduced Error Pruning Trees
RSS-ET	Random Subspace based Extra Tree
R	Correlation coefficient
SC	Soft computing
SC	Silt day Content
SVM	Support Vector Machine
NCHRP	National Cooperative Highway Research Program
LMBP	Levenberg–Marquardt Backpropagation
BP	Bayesian Programming
${\bar{y}}_{im}$	mean of measured values
*n*	Total number of data
*y_im_*	Measured values
*y_ip_*	Predicted values
